# Dysregulated Screen Exposure Is Associated With Severity of Language and Communication Impairments in Children With Developmental Disorders

**DOI:** 10.1111/apa.70223

**Published:** 2025-07-18

**Authors:** Andrea Hahnefeld, Monika Fink, Elena Hauber, Saskia Le Beherec, Marco Gassner, Matthias Klosinski, Volker Mall

**Affiliations:** ^1^ Chair of Social Pediatrics, TUM School of Medicine Technical University of Munich Munich Germany; ^2^ Kbo Kinderzentrum Munich Germany; ^3^ German Center for Child and Adolescent Health (DZKJ), Partner Site Munich Munich Germany

**Keywords:** developmental disorder, dysregulation, impairment, language, screen exposure

## Abstract

**Aim:**

While rising amounts of screen exposure for young children are reported, speech and language developmental disorders (DD) are also increasing. Vulnerable groups with combined risk factors are more affected, but less represented in the research. Our goal is to assess the amount and context of screen exposure in children referred for treatment and relate this to their language, cognitive and communication impairment.

**Methods:**

Retrospective analysis of medical records (parent ratings, child observations, standardised tests) for a random sample of 3–8‐year‐old children assessed by a multidisciplinary team of child experts at a social paediatric centre. Information about screen exposure in the home environment (screen exposure index, SEI), diagnoses and expert ratings were derived from reports and documentation.

**Results:**

Expert ratings yielded dysregulated screen exposure for 53% of the 73 children (mean age 4.7 years, SD 1.3, 82% male). Referred for suspected speech and language DD, 68% of the children were diagnosed with unspecific or mixed DD. Those with higher SEI ratings did not differ in IQ score, but received more unspecific or complex diagnoses and were rated as more severely impaired concerning language and communication.

**Conclusion:**

Screen exposure should be considered in diagnostic and therapeutic decisions.

**Abbreviations**
DD: (developmental disorder)
LCI: (Language Communication Index)
SEI: (Screen Exposure Index)

**Summary**

- Screen exposure is reported to be higher in vulnerable populations, but studies on sequelae are typically conducted on healthy children from voluntarily recruited families.
- In our clinical sample of 3–8‐year‐old children with developmental disorders, we found higher screen exposure to be significantly associated with more complex language and communication impairments.
- Screen exposure should be considered as a relevant environmental factor in assessing and treating children with developmental difficulties.

## 1 Introduction

Portable screens have become a regular part of everyday life. Current investigations report an average amount of screen time of 2 h per day for 2–4‐year‐olds and more than 3 h per day for 5–8‐year‐olds [1]. In lower‐income households, children are exposed to screens for twice as long as in higher‐income households [1]. The reported screen time exceeds expert recommendations [2, 3, 4] and replaces time for interpersonal interaction, active experiences and usage of skills in daily life [5, 6, 7]. In the literature, higher amounts of screen exposure at younger ages were associated with lower language and communication [8, 9, 10, 11] as well as less social interaction skills [5] and lower cognitive scores [11, 12, 13, 14, 15]. Parent–child talk has also been shown to decrease with increasing screen time in the home environment [5]. As children depend on interpersonal input to develop their language and communication skills [16], the amount of screen exposure in daily life might play a pivotal role, especially for children referred for clinical examinations due to language difficulties.

Specific developmental disorders (DD) of speech and language are defined as idiopathic, persistent problems in language development after the age of 3 years. According to diagnostic criteria, the disturbance of normal patterns of language acquisition from early stages is not attributable to neurological, perceptual, cognitive, or somatic conditions, or environmental factors [17]. Within the last few years, a rising amount of speech and language delays and disorders has been observed [18].

Research on the relation between language deficits and screen exposure has primarily analysed data on normally developing children based on surveys or experimentally recruited populations [5, 19, 20]. This approach tends to involve families with middle to high socioeconomic status and higher levels of education [5, 15, 21, 22]. Despite increased research activities in populations with lower socioeconomic status and children with weaker language performance [23], more research is needed on children with DD or from families with migration or multilingual backgrounds and less formal education, especially on those with combined risk factors [24].

High click‐rates and documented usage time of fast‐moving, low‐educational content especially designed for young children seem to suggest elevating rates of early‐age consumption world‐wide [25]. Corresponding algorithms to catch and maintain children's attention are constantly being refined [26]. In face of the complexity of the contemporary digital landscape [27], a comprehensive assessment of the children's screen exposure in everyday life is vital [28]. The amount of time as well as additional information on onset, access, frequency, content and supervision of screen activity should be considered in the measurement tools [8, 22, 27, 28]. Most studies have relied on parent rating as the only option available [29], although these assessments are liable to be inaccurate [30]. Parent ratings are also susceptible to memory effects, social desirability [28, 31] and distortions by caregivers' own media‐consumption habits [32, 33].

The aim of this study was to capture the amount and context of screen exposure and relate it to the language, communication and cognitive skills of children in a clinical population. Employing a multi‐method approach, we hypothesized that screen exposure was negatively associated with language and cognition scores and positively associated with language and communication impairments.

## 2 Patients and Methods

Using a clinical retrospective design, data for 73 randomly selected children were extracted from medical records and documentation in a social paediatric centre in Munich, Germany (kbo Kinderzentrum). All 3–8‐year‐old children that were referred to the social paediatric centre between 1 April and 30 June 2023 due to developmental difficulties in the areas of speech and language were eligible for the study. Those prediagnosed with early childhood disorders (like autism) or other serious somatic, neurological or psychiatric conditions affecting language development were excluded. The children and their primary caregivers had been assessed by a multidisciplinary team of specially trained physicians, psychologists and therapists. Information from the records was extracted by four different raters; for 23% of the children, a double analysis was performed for reliability coding.

In the centre, 2500 children per year affected by or at risk for DD are referred by their paediatricians for extensive multi‐professional diagnostic and treatment services. Medical reports are generated for clinical purposes and characterised by a high level of standardisation with predefined structures to generate multidimensional diagnoses, based on extensive use of standardised tests.

We assessed age, gender, languages spoken at home, and children's attendance at daycare or school as demographic variables. To estimate the socioeconomic status of the families, information on the parental level of education was extracted from questionnaires in the registration forms. We documented the amount of time children spent in front of screens in hours per day if reported by parents. We did not have exact specifications on time for each child, and information varied considerably across records. All available information on duration, type content and circumstances of screen exposure was extracted and summarised for each child. Subsequently, using a content‐structuring qualitative analysis [34], a deductive‐inductive combined approach based on important context information regarding screen time yielded five categories, which captured the most relevant dimensions in accordance with established measures [22, 28, 35] and recommendations from professional associations [2, 3, 4]. The Screen Exposure Index (SEI) comprises the absolute amount of screen time as well as information on access, frequency, type, age of onset, supervision and regulation of children's screen activities. We accounted for the variability of information in the medical records with defined anchoring points summarising several dimensions for each ordinally scaled category. The SEI was designed to avoid incomplete data due to non‐availability of exact screen exposure time specifications.

Category 0 was for children that had no screen exposure at all.

Category 1 comprised mild screen exposure limited to about 30 min per day and mainly educational media or entertainment when watching together as a family routine.

Category 2 was moderate screen exposure of about 1 h per day, background use, access to several devices or hard to regulate.

Category 3 was dysregulated screen exposure comprising times of up to 2 h per day, no parental regulations regarding screen exposure, or screens in demand as a constant companion in daily routines.

Category 4 was very dysregulated screen exposure of more than 2 h per day or with onset in early infancy. Further, when screens were described as a tool to comfort and regulate emotions for the child or as a permanent companion during daily routines, the child was classified in category 4.

The general and language impairment was assessed using the children's diagnoses of developmental disorders according to the 10th Revision of the International Classification of Diseases [17] in the medical records. To capture the amount of functional impairment in the cognitive domain, we referred to reported scores of standardised nonverbal and verbal IQ test scores. Additionally, to reflect the child's overall receptive and expressive abilities in their primary language as well as in German as their secondary language, we applied an exploratory approach. To capture language, communication, and interactions beyond the scope of the standardised measures, we created a severity index of clinically observed symptoms with information from the medical records. Similarly to the procedure for construction of the SEI, we analysed the content of the experts' descriptions to capture the child's level of verbal and non‐verbal communication abilities, employing a deductive –inductive combined approach [34] with a strong focus on clinical observations (Language Communication Index, LCI):

- Category 1 comprised children with minimal impairment, when only discrete deficits in one domain were described, for example, articulation disorder. Children in category 1 spoke in full sentences with light expressive or receptive difficulties, and reciprocal verbal communication was mostly age‐appropriate.
- Category 2 included children with mild impairment, defined as light deficits in communication in more than one domain, for example, not age‐appropriate vocabulary or dysgrammatism in expressive language. Despite mild deficits, reciprocal verbal communication was possible for children in this category.
- Category 3 was defined as moderate impairment. Children had apparent deficits in more than one domain, for example, limited vocabulary or not age‐appropriate expressive and receptive language skills. The child's age equivalence of language abilities was clearly below chronological age or the child was not always able to verbally express emotions and needs. Despite interest in interaction, reciprocal communication was difficult for children included in this category.
- Category 4 comprised children with severe impairment, defined as multiple deficits in several domains, for example, very limited vocabulary or at most short utterances with very low language comprehension. A child was classified in category 4 when it used words from languages spoken only in digital environments instead of words from languages spoken by the primary caregivers in the home environment. Additionally, when the age‐equivalence of language abilities was less than half of the chronological age, a child was classified in this category. Another criterion for the classification of a severe impairment was when the child's expressions were not understood by familiar and unfamiliar communication partners or the child showed abnormal communication and interaction behaviours. Verbal communication was almost impossible with children in category 4.
- Category 5 included children with very severe impairment, defined as profound deficits in all domains; for example, a child uttered almost no words at all or only sound imitations instead of words and did not react adequately to the verbal input or when being interacted with. When a child showed severe echolalia or the expressive language was described as monotonous and non‐modulated, and reciprocal verbal communication was not possible, it was classified in category 5.

The LCI was rated by two independent experts; where inconsistencies appeared, a third rater was consulted.

Retrospective analysis of the patient data was approved by the Ethics Committee of the Technical University of Munich, Faculty of Medicine, on 9 October 2023 (2023‐484‐S‐SB). The study was registered in the German Clinical Trials Register (DRKS) on 19 September 2024 (DRKS‐ID: DRKS00034197).

We calculated Spearman correlations and computed analysis of variances respectively tests for rank‐scaled data to represent between‐group differences for SEI with outcome variables, using IBM SPSS Statistics 28 (IBM Corp, New York, USA). A power analysis was conducted using G*Power (Version 3.1.9.7) [36].

## 3 Results

The majority (82%) of the 73 children of the study population was male, and 85% were raised in multilingual environments. Whereas 23% of the children attended regular education and childcare facilities, 60% were assigned to or visited special needs institutions, and 14% had been excluded from institutions due to behavioural problems. The mean age at first consultation in the social paediatric centre was 4 years and 8 months (see Table 1), and the children were accompanied by at least one primary caregiver. In the applications, 53% of mothers and 75% of fathers indicated information on education or current occupation. In total, 12% of mothers and 22% of fathers reported occupations with no specific education requirements, while 29% of mothers and 31% of fathers were involved in jobs demanding a non‐academic education like office clerk, baker, electrician or cook. Finally, 12% of mothers and 22% of fathers had professions associated with university degrees like physician, lawyer or engineer.

**TABLE 1:** Age at first consultation, amount of screen exposure and IQ scores of the study population.

		M	SD	Range	*N*
Age at first consultation (in years)		4.7	1.3	3.0–7.9	73
Amount of screen exposure (in h/day)		1.5	1.0	0.3–3.5	59
IQ scores	Composite	84	13	66–116	34
	Nonverbal	91	16	54–120	50
	Verbal comprehension	78	13	55–109	32
	Verbal acquisition	80	13	58–100	12

**FIGURE 1:**
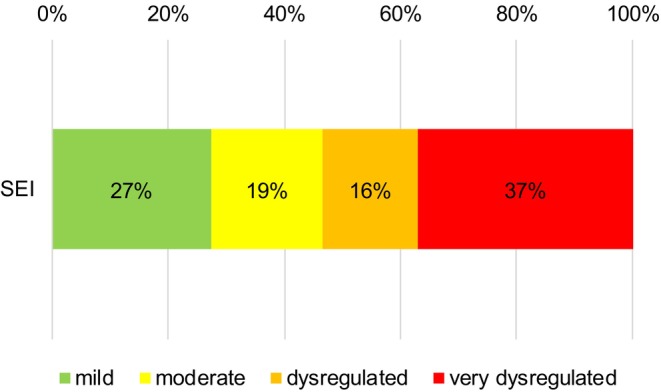
Screen exposure Index (SEI): Percentage of children per category.

The children's average daily screen exposure in parent ratings measured as an absolute amount of time was 1.5 h per day (Table 1). The results of the classification concerning SEI are depicted in Figure 1.

Screen exposure time and SEI showed a high and significant correlation (r_SET−SEI_ = 0.913, *p* < 0.001).

Diagnoses were recorded based on the International Classification of Diseases, 10th Revision (ICD‐10) [17]. The prevalence of the main diagnoses of the medical records is summarised in Table 2.

**TABLE 2:** Diagnoses with ICD‐10 codes of the study population extracted from the patients' records.

ICD‐10‐diagnoses (patients‘records)		
Developmental disorders (DD)		*N* (%)
F80 Speech and language	0.0 Specific speech articulation DD	1 (1)
	0.1 Expressive language DD	6 (8)
	0.2 Receptive language DD	15 (21)
F80.8/F80.9	Other/unspecified DD of speech and language	7 (10)
F83	Mixed specific DD	42 (58)
Other	Reading disorder (F81), no DD	2 (2)

For further analyses, diagnoses were divided into two categories, reflecting the complexity of the DD: Children with speech and language DD coded as specific speech articulation disorder (F80.0), expressive (F80.1), or receptive language disorder (F80.2) were grouped in one category labelled “no or language DD”, together with children that received no diagnosis of a DD. Children with other or unspecified language and mixed specific DD (F80.8, F80.9, F83) were grouped in another category labelled “unspecific or mixed DD”.

IQ test results were reported for 73% of the sample and are listed in Table 1. Of the remaining children, 16 were unable to complete standardised testing due to cooperation and attention difficulties.

**FIGURE 2:**
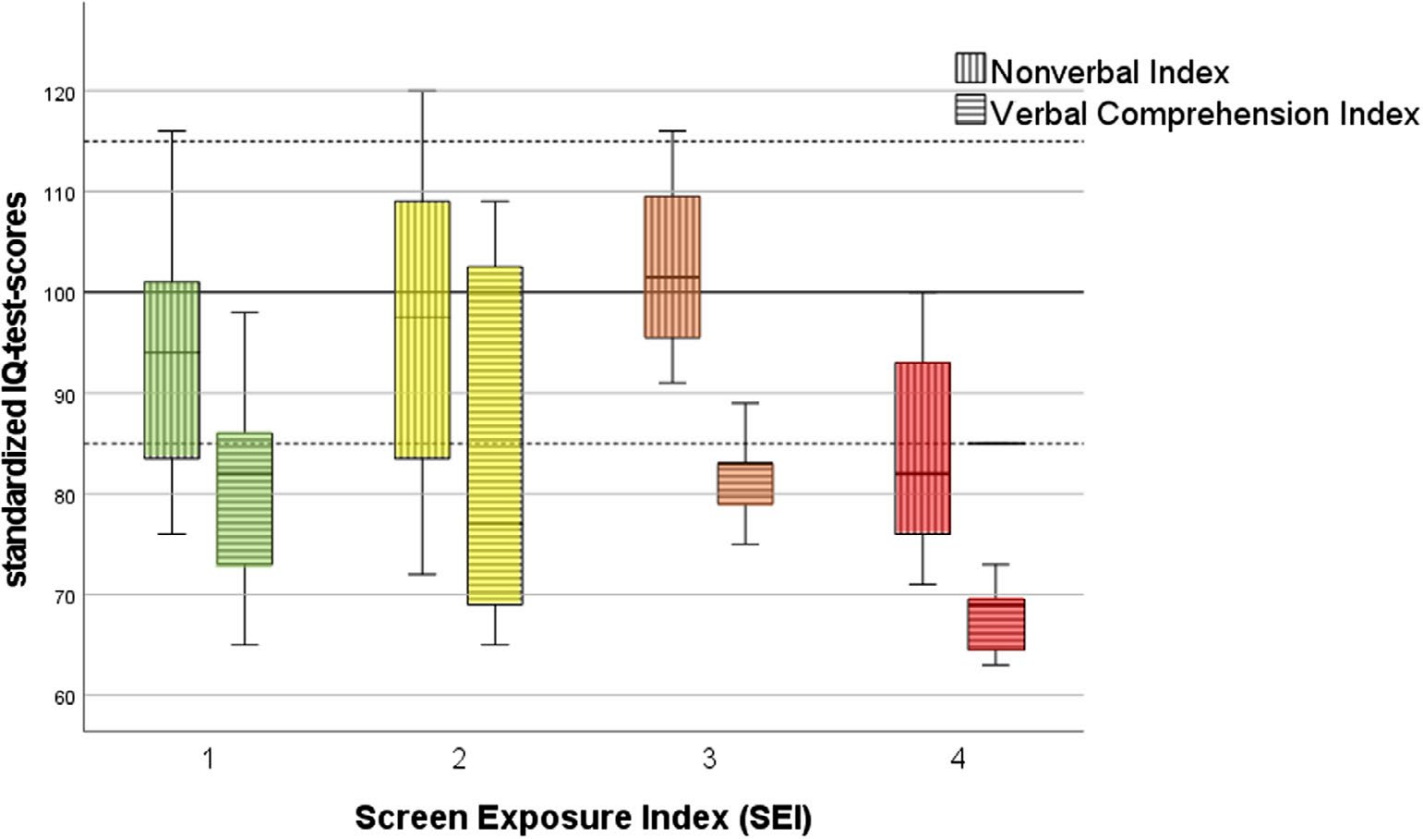
Distribution of nonverbal and verbal IQ scores in different categories of SEI, assessed with the Wechsler Preschool and Primary Scale of Intelligence‐IV (WIPPSI‐IV, 64%), Wechsler Nonverbal Scale of Ability (WNV, 15%), Kaufman Assessment Battery for Children‐II (KABC‐II, 8%), Snijders‐Oomen Nonverbal Intelligence Test (SON‐R 2–8, 8%), and the Wechsler Intelligence Scale for Children‐V (WISC‐V, 6%).

In our sample, the mean nonverbal cognitive indices were located within the normal range, whereas all mean indices involving language scores fell below average; see Table 1 and Figure 2.

**FIGURE 3:**
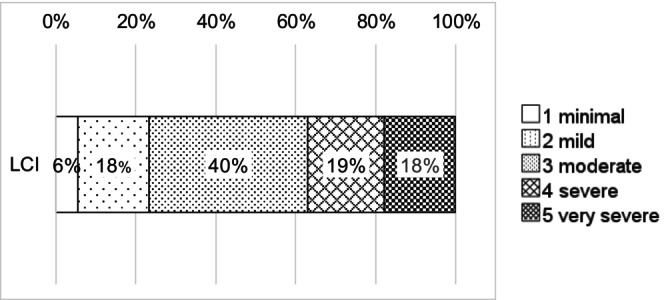
Language‐Communication‐Index (LCI): Level of impairment, percentage of children per category.

The results of our classification for the LCI are in Figure 3. The rater agreed in 73% of the decisions. We carried out an inter‐rater reliability analysis with the calculation of Weighted Cohen's Kappa and found good agreement between the samples of rater 1 and rater 2 with *κ* = 0.78.

**FIGURE 4:**
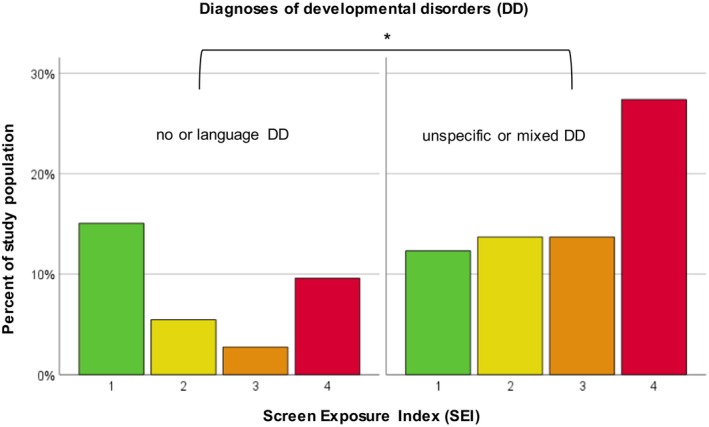
Distribution of different categories of Screen Exposure Index (SEI) among diagnoses of developmental disorders (DD).

Children in the group “unspecific language or mixed DD” showed a significantly higher SEI than those grouped in “no or language DD” (Mann‐Whitney *U* = 426.50, *p* = 0.048), see Figure 4. With a calculated sample size of 52 (26 per group) and group proportions of 0.67 for “unspecific language or mixed DD” and 0.33 for “no or language DD”, our analysis yielded a power of 0.80 for a medium effect.

An analysis of variance showed no significant differences for nonverbal or verbal IQ test scores in relation to SEI (FSEI‐NVI (3,46) = 1.759, *p* = 0.168; FSEI‐VCI (3, 28) = 2.763, *p* = 0.061), see Figure 2.

**FIGURE 5:**
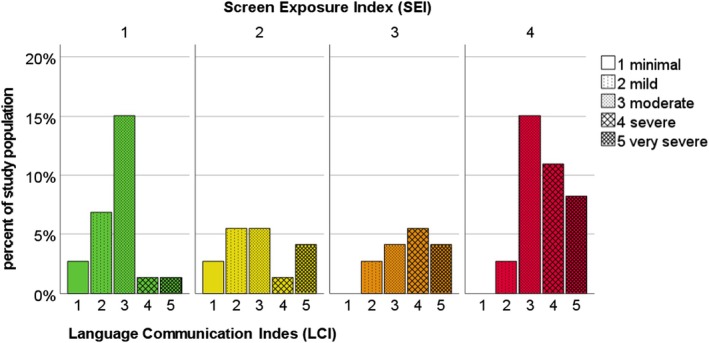
Distribution of expert rated Language Communication Index (LCI) in different categories of Screen Exposure Index (SEI).

Children with a higher SEI demonstrated more profound deficits in language and communication as rated on the LCI (Kruskal–Wallis H (3) = 11.854, *p* = 0.008), see Figure 5.

For chi‐squared test with a medium effect size of *ρ* = 0.381, a significance level of *α* = 0.05 (one‐tailed) and a sample size of 73 participants, the actual power achieved was 0.901.

Children with “no or language DD” had lower LCI ratings than children diagnosed with “unspecific or mixed DD” (H (1) = 5.080, *p* = 0.024). For children with both assessments, the LCI showed a significantly negative association with IQ scores (r_LCI‐nonverbal IQ_ = −0.387, *p* = 0.006, *n* = 50; r_LCI‐verbal IQ_ = −0.414, *p* = 0.019, *n* = 32), whereas IQ scores did not differ between the diagnostic categories (t_Dia‐nonverbal IQ_ (48) = 1.821, *p* = 0.075; t_Dia‐verbal IQ_ (30) = −0.011, *p* = 0.991).

## 4 Discussion

In this study, we show that higher ratings of screen exposure in our clinical sample were associated with more complex and unspecific symptoms, deficits and diagnoses. With a multi‐method approach, we captured high rates of dysregulated screen exposure and the children's real‐life language and communication impairments in a hard‐to‐reach population. The combination of standardised test results with parent and expert ratings reveals that a high proportion of the children did not display isolated speech and language DD or cognitive deficits but noticeable interaction difficulties in various environments.

Our results demonstrate that all children were exposed to screens in daily life and 73% exceeded the German and 50% the American screen exposure time guidelines [2, 3, 4], which is in line with previous research [1]. The average amount of screen time per day in our sample was in the lower range compared to data from comparable age groups in the most recent studies [1].

More than two thirds of the children presented with suspected language development difficulties were diagnosed with unspecified language or mixed DD. This residual category (F83) comprises disorders in which there is a mixture or overlap of two or more categories of circumscribed DDs like speech and language, school skills and motor functions, often combined with some degree of general impairment in cognitive functioning [17]. In our sample, we did not find any relations between IQ scores and diagnostic categories, but more complex symptom patterns involving several areas of development were associated with higher SEI ratings. This highlights the importance of also considering different question formats and contextual information in multi‐professional paediatric assessment. Daily routines can either act as positive opportunities for interpersonal communication or reinforce withdrawal by offering secluded digital entertainment. Consequently, young children might suffer from over‐stimulation due to screen exposure as well as lack of comforting interaction with their primary caregivers [8]. If screens are used as a tool to manage children's emotions in daily life, they might even replace parental co‐regulation as a necessary experience and lead to dysfunctional dependencies in later life [33].

The diagnostic categories of unspecified or mixed developmental disorders do not explicitly define the domains and severity of the symptoms. The additional consideration of the IQ scores and the creation of the LCI provide valuable information to capture children's real‐life deficits. The fact that mean nonverbal IQ scores remained within the normal range further highlights the severity of the impairments in supporting functions like frustration tolerance, openness to stimuli, and behaviour regulation in complex social environments. The below‐average verbal IQ scores might reflect underlying language and knowledge deficits associated with screen exposure. On the other hand, these results could be explained by the fact that a large proportion of the children had been assessed in German as their second language. Importantly, 22% of the children were unable to complete standardised IQ assessments, which may lead to an underestimation of the problem.

## 5 Limitations

The lack of a control group without language deficits and the high variability of the sample regarding age and socioeconomic status limits the generalisability of the results. Nevertheless, by comparing age‐corrected standard values of test scores, balancing information from the test situation and home environment, and incorporating expert ratings, we accounted for the heterogeneity of our sample.

Despite the comparison with standardised test scores for cognitive development, a limitation of our study is the lack of objective tools to assess screen exposure. We referred to reports and indirect information from the records which comprised detailed descriptions of a child's daily routines and activities, including eating and sleeping habits. This data revealed important specifications about the time and context of screen exposure beyond parents' estimation of screen time.

Although we defined anchor points within the scales, we did not explicitly state how SEI and LCI criteria were weighted in the expert ratings. Whereas extended time with low‐quality media is known to have negative effects, screens might be helpful in maintaining contact with family members abroad and offer valuable interaction and learning opportunities. A mediating factor for developmental effects might be the purpose of children's screen use, which we refer to at the two endpoints of our SEI scale. Accordingly, a growing body of research is focusing on education and connection purpose versus regulatory media use in parents and children [23, 33]. Due to the retrospective character of the analysis, we were not able to disentangle the entities of time and type of use or to evaluate intentions in our sample. This approach allows for deviations due to individual interpretations and calls for validations of the scales with larger samples and experts from different cultural and professional backgrounds.

## 6 Strengths

A strength of this study is the multi‐method interdisciplinary assessment of the children's real‐life impairment in the cognitive, language and communication domain by combining extensive assessments from clinical experts with standardised test results. With our clinical sample, we reached a group with combined risk factors from various socioeconomic backgrounds that might be underrepresented in studies thus far due to insufficient German language skills for large‐scale questionnaires [28]. While other studies often exclude participants from special needs educational facilities [37], in our study three quarters did not attend regular schools. Whereas the level of parental education in previous studies is rather high with more than half of the mothers having an academic degree [5, 10, 11, 21], only 12% of the mothers in our sample reported jobs requiring university education. Many studies include only children from families where the dominant language is the national language [19, 22, 37]; however, in our sample most of the children were raised in multilingual environments with German not the dominant language. Due to the cross‐sectional study design, our finding that screen exposure correlated significantly with the level and severity of children's developmental difficulties does not prove a causal connection. The possibility that children were exposed to screens because of their challenging behaviours or to compensate for communication difficulties as part of DD has to be considered.

## 7 Conclusion

Environmental influences must be taken into account in child assessment and treatment, as higher dysregulation of screen exposure in our clinical sample was significantly associated with more severe impairments in language and social‐interactive abilities.

## Author Contributions

**Andrea Hahnefeld:** conceptualization, funding acquisition, writing – original draft, methodology, validation, visualization, writing – review and editing, formal analysis, project administration, data curation, supervision, resources, investigation. **Monika Fink:** conceptualization, investigation, writing – original draft, methodology, validation, writing – review and editing, formal analysis, data curation, visualization. **Elena Hauber:** conceptualization, investigation, writing – original draft, methodology, validation, writing – review and editing, supervision. **Saskia Le Beherec:** conceptualization, investigation, methodology, validation, writing – review and editing, formal analysis, data curation, supervision. **Marco Gassner:** writing – original draft, methodology, validation, visualization, writing – review and editing, software. **Matthias Klosinski:** supervision, resources, project administration, validation, conceptualization, investigation, writing – review and editing. **Volker Mall:** conceptualization, funding acquisition, methodology, validation, writing – review and editing, project administration, supervision.

## Conflicts of Interest

The authors declare no conflicts of interest.

## References

[1] S. Mann , A. Calvin , A. Lenhart , et al., “The Common Sense Census: Media Use by Kids Zero to Eight,” San Francisco, CA: Common Sense Media, https://www.commonsensemedia.org/sites/default/files/research/report/2025‐common‐sense‐census‐web‐2.pdf.

[2] AAP Council on communications and media , “Media and Young Minds,” Pediatrics 138 (2016): e20162591. 27940793 10.1542/peds.2016-2591

[3] World Health Organization , Guidelines on Physical Activity, Sedentary Behaviour, and Sleep for Children Under 5 Years of Age (World Health Organization, 2019). 31091057

[4] Deutsche Gesellschaft für Kinder‐ und Jugendmedizin e.V. DGKJ , “SK2‐Leitlinie: Leitlinie Zur Prävention Dysregulierten Bildschirmmediengebrauchs in der Kindheit und Jugend (AWMF‐Register Nr. 027–075),” https://register.awmf.org/de/leitlinien/detail/027‐075.

[5] M. E. Brushe , D. G. Haag , E. C. Melhuish , S. Reilly , and T. Gregory , “Screen Time and Parent‐Child Talk When Children Are Aged 12 to 36 Months,” JAMA Pediatrics 178 (2024): 369–375. 38436942 10.1001/jamapediatrics.2023.6790 PMC10913002

[6] S. Madigan , D. Browne , N. Racine , C. Mori , and S. Tough , “Association Between Screen Time and Children's Performance on a Developmental Screening Test,” JAMA Pediatrics 173 (2019): 244–250. 30688984 10.1001/jamapediatrics.2018.5056 PMC6439882

[7] E. Swider‐Cios , A. Vermeij , and M. M. Sitskoorn , “Young Children and Screen‐Based Media: The Impact on Cognitive and Socioemotional Development and the Importance of Parental Mediation,” Cognitive Development 66 (2023): 101319.

[8] M. L. Jara Baquerizo , K. A. Mayorga Arias , and N. S. Reyes Lozano , “Pantallas y Adquisición Del Lenguaje: ¿Cuánto Tiempo es Demasiado?,” Reincisol 3 (2024): 6796–6820.

[9] S. Madigan , B. A. McArthur , C. Anhorn , R. Eirich , and D. A. Christakis , “Associations Between Screen Use and Child Language Skills: A Systematic Review and Meta‐Analysis,” JAMA Pediatrics 174 (2020): 665–675. 32202633 10.1001/jamapediatrics.2020.0327 PMC7091394

[10] H. Byeon and S. Hong , “Relationship Between Television Viewing and Language Delay in Toddlers: Evidence From a Korea National Cross‐Sectional Survey,” PLoS One 10 (2015): e0120663. 25785449 10.1371/journal.pone.0120663 PMC4365020

[11] J. S. Hutton , J. Dudley , T. Horowitz‐Kraus , T. DeWitt , and S. K. Holland , “Associations Between Screen‐ Based Media Use and Brain White Matter Integrity in Preschool‐Aged Children,” JAMA Pediatrics 174 (2020): e193869. 31682712 10.1001/jamapediatrics.2019.3869 PMC6830442

[12] K. Kostyrka‐Allchorne , N. R. Cooper , and A. Simpson , “The Relationship Between Television Exposure and Children's Cognition and Behaviour: A Systematic Review,” Developmental Review 44 (2017): 19–58.

[13] R. Aishworiya , S. Cai , H. Y. Chen , et al., “Television Viewing and Child Cognition in a Longitudinal Birth Cohort in Singapore: The Role of Maternal Factors,” BMC Pediatrics 19 (2019): 286. 31419962 10.1186/s12887-019-1651-z PMC6696668

[14] F. W. Paulus , E. Möhler , F. Recktenwald , A. Albert , and V. Mall , “Electronic Media and Early Childhood: A Review,” Klinische Pädiatrie 233 (2021): 157–172. 33662997 10.1055/a-1335-4936

[15] B. Guellai , E. Somogyi , R. Esseily , and A. Chopin , “Effects of Screen Exposure on Young Children's Cognitive Development: A Review,” Frontiers in Psychology 13 (2022): 13. 10.3389/fpsyg.2022.923370 PMC9431368 36059724

[16] A. Sundqvist , F.‐S. Koch , U. Birberg Thornberg , R. Barr , and M. Heimann , “Growing up in a Digital World ‐ Digital Media and the Association With the Child's Language Development at Two Years of Age,” Frontiers in Psychology 12 (2021): 12. 10.3389/fpsyg.2021.569920 PMC8015860 33815187

[17] World Health Organization , “International Statistical Classification of Diseases and Related Health Problems 10th Revision (ICD‐10)‐WHO Version for; 2019‐ Covid‐Expanded,” https://icd.who.int/browse10/2019/en.

[18] T. Khan , R. Freeman , and A. Druet , “Komodo Health. Louder Than Words: Pediatric Speech Disorders Skyrocket Throughout Pandemic, 2023”, https://www.komodohealth.com/hubfs/2023/Speech_Pathology_Research_Brief. pdf?hsCtaTracking=e40f8354‐b93c‐4306‐9160‐2bcce2f146de%7Cf835bb14.

[19] D. A. Christakis , J. Gilkerson , J. A. Richards , et al., “Audible Television and Decreased Adult Words, Infant Vocalizations, and Conversational Turns: A Population‐Based Study,” Archives of Pediatrics & Adolescent Medicine 163 (2009): 554–558. 19487612 10.1001/archpediatrics.2009.61

[20] M. Jing , T. Ye , H. L. Kirkorian , and M. L. Mares , “Screen Media Exposure and Young Children's Vocabulary Learning and Development: A Meta‐Analysis,” Child Development 94 (2023): 1398–1418. 37042116 10.1111/cdev.13927

[21] M. M. Hedderson , T. A. Bekelman , M. Li , et al., “Trends in Screen Time Use Among Children During the COVID‐19 Pandemic, July 2019 Through August 2021,” JAMA Network Open 6 (2023): e2256157. 36790805 10.1001/jamanetworkopen.2022.56157 PMC9932850

[22] J. S. Hutton , G. Huang , R. D. Sahay , T. DeWitt , and R. F. Ittenbach , “A Novel, Composite Measure of Screen‐ Based Media Use in Young Children (ScreenQ) and Associations With Parenting Practices and Cognitive Abilities,” Pediatric Research 87 (2020): 1211–1218. 32050256 10.1038/s41390-020-0765-1

[23] S. C. Kucker , L. K. Perry , and R. Barr , “Variability and Patterns in Children's Media Use and Links With Language Development,” Acta Paediatrica 113 (2024): 1032–1039. 38197331 10.1111/apa.17100 PMC11006579

[24] B. T. McDaniel , L. Linder , M. M. P. van den Abeele , et al., “Technoference in Parenting and Impacts on Parent–Child Relationships and Child Development,” in Handbook of Children and Screens, ed. D. A. Christakis and L. Hale (Springer Nature Switzerland, 2025), 411–417.

[25] Y. L. Reid Chassiakos , J. Radesky , D. Christakis , M. A. Moreno , and C. Cross , “Children and Adolescents and Digital Media,” Pediatrics 138 (2016): e20162593. 27940795 10.1542/peds.2016-2593

[26] J. Radesky , E. Bridgewater , S. Black , et al., “Algorithmic Content Recommendations on a Video‐Sharing Platform Used by Children,” JAMA Network Open 7 (2024): e2413855. 38809550 10.1001/jamanetworkopen.2024.13855 PMC11137630

[27] R. Byrne , C. O. Terranova , and S. G. Trost , “Measurement of Screen Time Among Young Children Aged 0‐6 Years: A Systematic Review,” Obesity Reviews 22 (2021): e13260. 33960616 10.1111/obr.13260 PMC8365769

[28] R. Barr , H. Kirkorian , J. Radesky , et al., “Beyond Screen Time: A Synergistic Approach to a More Comprehensive Assessment of Family Media Exposure During Early Childhood,” Frontiers in Psychology 11 (2020): 1283. 32754078 10.3389/fpsyg.2020.01283 PMC7365934

[29] H. Duch , E. M. Fisher , I. Ensari , and A. Harrington , “Screen Time Use in Children Under 3 Years Old: A Systematic Review of Correlates,” International Journal of Behavioral Nutrition and Physical Activity 10 (2013): 102. 23967799 10.1186/1479-5868-10-102 PMC3844496

[30] J. S. Radesky , H. M. Weeks , R. Ball , et al., “Young Children's Use of Smartphones and Tablets,” Pediatrics 146 (2020). 10.1542/peds.2019-3518 PMC7329252 32482771

[31] M. H. Bornstein , D. L. Putnick , J. E. Lansford , et al., “Mother and Father Socially Desirable Responding in Nine Countries: Two Kinds of Agreement and Relations to Parenting Self‐Reports,” International Journal of Psychology 50 (2015): 174–185. 25043708 10.1002/ijop.12084 PMC4297254

[32] J. Zhang , Q. Zhang , B. Xiao , Y. Cao , Y. Chen , and Y. Li , “Parental Technoference and Child Problematic Media Use: Meta‐Analysis,” Journal of Medical Internet Research 27 (2025): e57636. 39841982 10.2196/57636 PMC11799820

[33] B. Suh , H. Kirkorian , R. Barr , S. C. Kucker , C. Torres , and J. S. Radesky , “Measuring Parents' Regulatory Media Use for Themselves and Their Children,” Frontiers in Developmental Psychology 2 (2024): 2. 10.3389/fdpys.2024.1377998 PMC12758898 41487697

[34] U. Kuckartz and S. Rädiker , Qualitative Inhaltsanalyse. Methoden, Praxis, Umsetzung Mit Software und künstlicher Intelligenz (Juventa Verlag, 2024).

[35] H. Klakk , C. T. Wester , L. G. Olesen , et al., “The Development of a Questionnaire to Assess Leisure Time Screen‐Based Media Use and Its Proximal Correlates in Children (SCREENS‐Q),” BMC Public Health 20 (2020): 664. 32397984 10.1186/s12889-020-08810-6 PMC7216486

[36] F. Faul , E. Erdfelder , A. Buchner , et al., “Statistical Power Analyses Using G*Power 36.1: Tests for Correlation and Regression Analyses,” Behavior Research Methods 41 (2009): 1149–1160. 19897823 10.3758/BRM.41.4.1149

[37] C. F. Norbury , D. Gooch , C. Wray , et al., “The Impact of Nonverbal Ability on Prevalence and Clinical Presentation of Language Disorder: Evidence From a Population Study,” Journal of Child Psychology and Psychiatry 57 (2016): 1247–1257. 27184709 10.1111/jcpp.12573 PMC5082564

